# Should dual antiplatelet therapy be used in patients following coronary artery bypass surgery? A meta-analysis of randomized controlled trials

**DOI:** 10.1186/s12893-015-0096-z

**Published:** 2015-10-14

**Authors:** Subodh Verma, Shaun G. Goodman, Shamir R. Mehta, David A. Latter, Marc Ruel, Milan Gupta, Bobby Yanagawa, Mohammed Al-Omran, Nandini Gupta, Hwee Teoh, Jan O. Friedrich

**Affiliations:** Division of Cardiac Surgery, Keenan Research Centre for Biomedical Science and Li Ka Shing Knowledge Institute of St. Michael’s Hospital, Toronto, ON M5B 1W8 Canada; Division of Cardiology, Li Ka Shing Knowledge Institute of St. Michael’s Hospital, Toronto, ON M5B 1W8 Canada; Division of Vascular Surgery, Keenan Research Centre for Biomedical Science and Li Ka Shing Knowledge Institute of St. Michael’s Hospital, Toronto, ON M5B 1W8 Canada; Division of Endocrinology & Metabolism, Keenan Research Centre for Biomedical Science and Li Ka Shing Knowledge Institute of St. Michael’s Hospital, Toronto, ON M5B 1W8 Canada; Department of Surgery, St. Michael’s Hospital, Toronto, ON M5B 1W8 Canada; Department of Medicine, St. Michael’s Hospital, Toronto, ON M5B 1W8 Canada; Department of Critical Care, St. Michael’s Hospital, Toronto, ON M5B 1W8 Canada; Department of Surgery, University of Toronto, Toronto, ON M5S 2J7 Canada; Department of Medicine, University of Toronto, Toronto, ON M5S 2J7 Canada; Interdepartmental Division of Critical Care, University of Toronto, Toronto, ON M5S 2J7 Canada; King Saud University, Riyadh, 12372 Saudi Arabia; Department of Medicine, McMaster University, Hamilton, ON L8L 2X2 Canada; Population Health Research Institute, Hamilton Health Sciences, Hamilton, ON L8L 2X2 Canada; University of Ottawa Heart Institute, Ottawa, ON K1Y 4W7 Canada; Canadian Cardiovascular Research Network, Brampton, ON L6Z 4N5 Canada; The Ottawa Hospital, Ottawa, ON K1H 8L6 Canada

**Keywords:** Coronary artery bypass graft surgery, Acute coronary syndrome, Anti-platelet therapy, P2Y_12_ antagonists, Systematic review, Meta-analysis

## Abstract

**Background:**

We assessed the effectiveness of dual antiplatelet therapy (DAPT) post elective or urgent (i.e., post acute coronary syndrome [ACS]) coronary artery bypass graft surgery (CABG).

**Methods:**

We systematically searched MEDLINE, EMBASE, and the Cochrane Registry from inception to August 2015. Randomized controlled trials (RCTs) in adults undergoing CABG comparing either dual vs. single antiplatelet therapy or higher- vs. lower-intensity DAPT were identified.

**Results:**

Nine RCTs (*n* = 4,887) with up to 1y follow-up were included. Five RCTs enrolled patients post-elective CABG (*n* = 986). Two multi-centre RCTs enrolled ACS patients who subsequently underwent CABG (*n* = 2,155). These 7 RCTs compared clopidogrel plus aspirin to aspirin alone. Two other multi-centre RCTs reported on ACS patients who subsequently underwent CABG comparing higher intensity DAPT with either ticagrelor (*n* = 1,261) or prasugrel (*n* = 485) plus aspirin to clopidogrel plus aspirin. Post-operative anti-platelet therapy was started when chest tube bleeding was no longer significant, typically within 24–48 h. There were no differences in all-cause mortality in clopidogrel plus aspirin vs. aspirin RCTs; conversely, all-cause mortality was significantly lower in ticagrelor and prasugrel vs. clopidogrel RCTs (risk ratio[RR] 0.49, 95 % confidence interval[CI] 0.33–0.71, *p* = 0.0002; 2 RCTs, *n* = 1695; *I*^*2*^ = 0 %; interaction *p* < 0.01 compared to clopidogrel plus aspirin vs aspirin RCTs). There were no differences in myocardial infarctions, strokes, or composite outcomes. Overall, major bleeding was not significantly increased (RR 1.31, 95 % CI 0.81–2.10, *p* = 0.27; 7 RCTs, *n* = 4500). There was heterogeneity (*I*^*2*^ *=* 42 %) due almost entirely to higher bleeding reported for the prasugrel RCT which included mainly CABG-related major bleeding (RR 3.15, 95 % CI 1.45–6.87, *p* = 0.004; 1 RCT, *n* = 437).

**Conclusions:**

Most RCT data for DAPT post CABG is derived from subgroups of ACS patients in DAPT RCTs requiring CABG who resume DAPT post-operatively. Limited RCT data with heterogeneous trial designs suggest that higher intensity (prasugrel or ticagrelor) but not lower intensity (clopidogrel) DAPT is associated with an approximate 50 % lower mortality in ACS patients who underwent CABG based on post-randomization subsets from single RCTs. Large prospective RCTs evaluating the use of DAPT post-CABG are warranted to provide more definitive guidance for clinicians.

**Electronic supplementary material:**

The online version of this article (doi:10.1186/s12893-015-0096-z) contains supplementary material, which is available to authorized users.

## Background

Dual antiplatelet therapy (DAPT) with acetylsalicylic acid (ASA) and an oral P2Y_12_ antagonist, is the cornerstone for management of patients presenting with acute coronary syndrome (ACS). Although there are no recommendations regarding DAPT treatment post elective CABG, guidelines recommend that therapy with DAPT be continued for one year following ACS (non-ST segment elevation myocardial infarction [NSTEMI], ST-elevation myocardial infarction [STEMI] or unstable angina), irrespective of whether patients are managed medically or invasively with either percutaneous coronary intervention or coronary artery bypass graft (CABG) surgery [1, 2]. The evidence supporting the benefit of DAPT in ACS patients has been based largely on three landmark trials, namely CURE [3], TRITON-TIMI 38 [4] and PLATO [5]. Whereas cardiac surgeons are well versed with the guidelines regarding discontinuation of DAPT prior to CABG to minimize bleeding risks [6, 7], there is considerable variability in DAPT resumption in ACS patients post CABG [8–10]. Since only a small proportion of patients in large trials undergo CABG, individual RCTs have not been adequately powered to address the role of DAPT in the post-CABG cohort. We therefore, conducted a meta-analysis of RCTs to evaluate the benefits of DAPT resumption in post CABG patients, either elective or post ACS, using all-cause mortality as the pre-specified primary endpoint.

## Methods

### Data sources

**Fig. 1:**
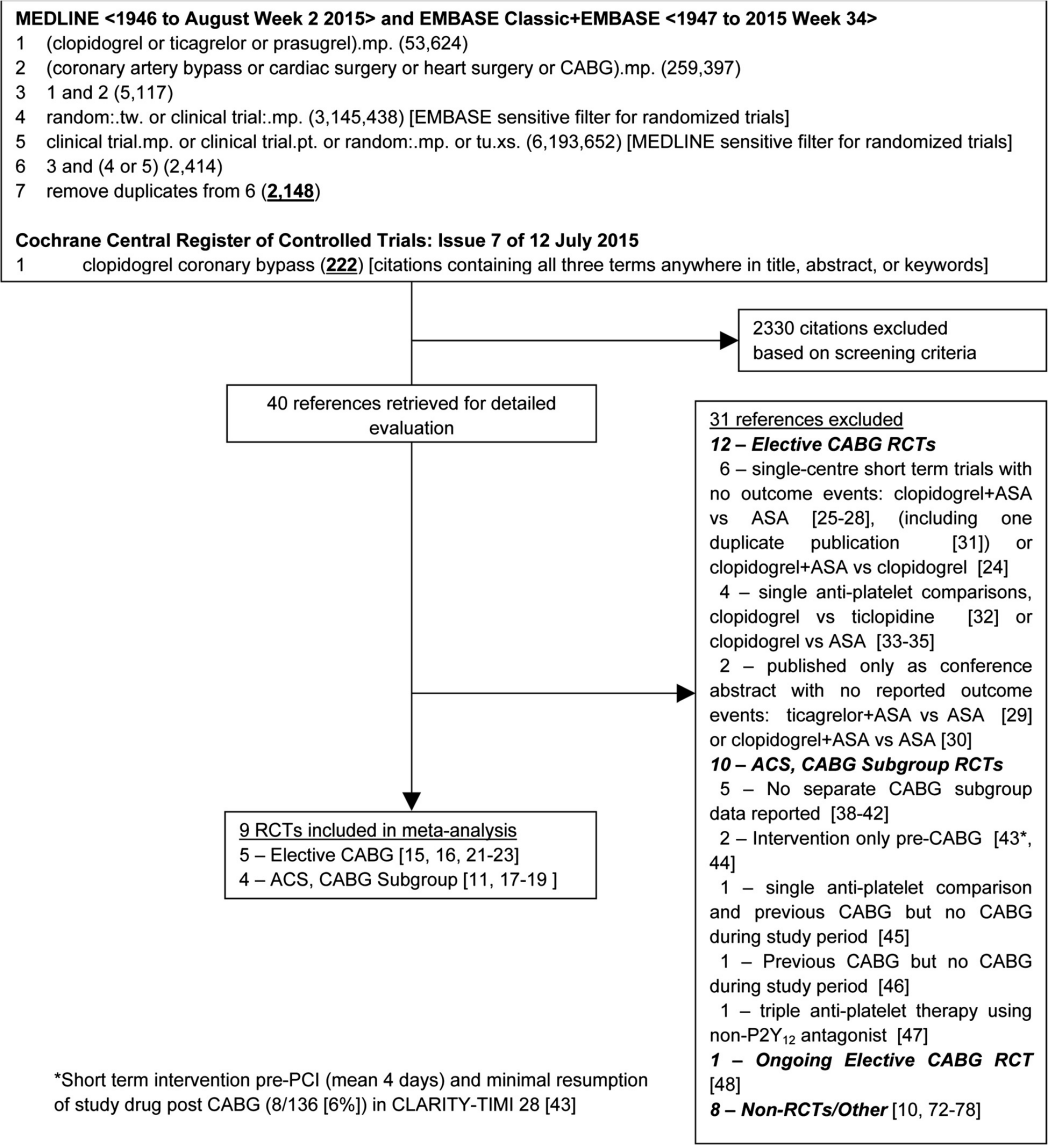
Search strategy and trial flow. Flow chart for the systematic review and meta-analysis showing the search strategy, and the number of studies retained and number of studies excluded with reason for exclusion at each stage of the study selection process [72–78]

We systematically searched OVID versions of MEDLINE (1946 through to August 2015, week 2), EMBASE Classic and EMBASE (1947 through 2015 week 34), and the Cochrane Central Register of Controlled Trials (July 2015) for relevant studies using search terms for anti-platelet agents and coronary artery bypass surgery, and published sensitive filters to identify randomized controlled trials (RCTs) (Fig. 1). We also searched bibliographies of included studies and personal files. We did not impose language restrictions.

### Study Selection

We included prospective clinical trials randomizing adult patients with coronary artery disease undergoing CABG either 1) to dual vs single antiplatelet therapy (e.g., clopidogrel plus ASA vs ASA alone) or 2) to higher vs lower intensity dual antiplatelet therapy (e.g., ticagrelor or prasugrel plus ASA vs clopidogrel plus ASA). To meet inclusion criteria, RCTs had to either include only patients undergoing CABG, or report outcomes separately in subgroups of patients with ACS who underwent CABG. Studies were excluded if patients were not randomized between treatment groups or if outcomes were not reported separately for patients undergoing CABG. Citations were screened in duplicate and full text review was conducted to determine eligibility when either screening reviewer felt a citation potentially met inclusion criteria.

### Data Extraction and Quality Assessment

Details of the publication (i.e., trial authors and acronym, enrolment period, year of publication), inclusion/exclusion criteria, demographics and cardiac risk factors of the enrolled patients, description of the interventions used, and outcome definitions and events were collected and collated. Risk of bias in RCTs (including blinding of participants, method of sequence generation and allocation concealment, intention-to-treat analysis, early trial stopping for efficacy before the planned enrollment was completed, and loss to follow-up) was also assessed.

### Data Analysis

Our primary hypothesis was that all-cause mortality at the longest duration of follow up is decreased in patients undergoing CABG randomized to dual antiplatelet therapy vs patients randomized to lower intensity dual or single antiplatelet therapy. Secondary outcomes, also at the longest duration of follow up, included non-fatal myocardial infarction (MI); non-fatal stroke; the composite outcome of cardiovascular (alternatively all-cause) mortality or non-fatal MI or non-fatal stroke; and bleeding (major bleeding, preferably non-CABG related, if reported, otherwise all major bleeding). The primary analysis included all patients in an intention to treat analysis. Prespecified subgroup analyses were conducted comparing 1) dual vs single antiplatelet therapy trials in elective CABG patients, to 2) dual vs single antiplatelet therapy trials in ACS patients who underwent CABG, to 3) higher intensity vs lower intensity dual antiplatelet trials in ACS patients who underwent CABG. We planned to also conduct a supplementary “on treatment” analysis including only patients who were actually continued on study drugs post-operatively, but these data were only available for one outcome (mortality) for one trial [11]. All meta-analyses were performed using Review Manager (RevMan version 5.2; Cochrane Collaboration, Oxford, UK) by one of the authors (JOF). Random effects models [12] which incorporate between-trial heterogeneity and give wider and more conservative confidence intervals (CI) when heterogeneity is present were used for all analyses. Statistical heterogeneity among trials was assessed using *I*^*2*^, defined as the percentage of total variability across studies attributable to heterogeneity rather than chance, and using published guidelines for low (*I*^*2*^ = 25 %–49 %), moderate (*I*^*2*^ = 50 %–74 %) and high (*I*^*2*^ ≥ 75 %) heterogeneity [13]. Risk ratios (RR) were used to pool outcomes with a two-sided significance level of 5 %. Individual trial and summary results are reported with 95 % CIs. Differences between pooled RRs were evaluated using z tests. To assess for publication bias, a funnel plot comparing effect measure for the primary outcome of mortality to study precision was examined for evidence of asymmetry. Further more formal statistical testing for funnel plot asymmetry was not carried out due to the low number of RCTs meeting inclusion criteria (<10) which would result in low statistical power to distinguish chance from real asymmetry [14].

We contacted authors of included trials to clarify outcome data when required. Authors of one trial provided the numbers of patients who sustained myocardial infarctions and informed us that rates of stroke were not measured [15]. For the remainder we were informed that no additional data were available [11, 16–18]. For two ACS trials only the composite outcome of either cardiovascular [19] or all-cause mortality [18], MI, and stroke were reported for the subgroup of patients who underwent CABG. Authors of one of these trials provided individual event rates [19]. For the other trial, we estimated all-cause mortality, MI, and stroke rates assuming the same overall ratios of the separate outcomes to the composite outcome for each randomized group as reported in the main trial publication [20].

## Results

The initial search strategy yielded 2148 citations from MEDLINE and EMBASE, and 222 citations from Cochrane, of which 40 were retrieved for full text review. Nine RCTs met inclusion criteria (Fig. 1) [11, 15–19, 21–23]. Details regarding excluded RCTs are provided in Additional file 1: Table S1. In brief, RCTs in patients post elective CABG were excluded if no outcome events occurred or were reported [24–30] (including duplicate publication [31]) or single anti-platelet therapies were used in both randomized groups [32–35] (including duplicate publications [36, 37]). RCTs enrolling patients with ACS were excluded if no separate outcome data was available for the subgroup of patients who underwent CABG (CHARISMA [38], DISPERSE-2 [39], COMMIT [40], TRILOGY ACS [41], and JUMBO-TIMI 26 [42]), patients were not treated with dual-antiplatelet therapy post CABG (CLARITY-TIMI 28 [43] and CURRENT OASIS 7 [44]), trials reported only on patients previously treated with CABG but not during the study period (CAPRIE [45] and PLATO [46]); or patients were randomized to a non-P2Y_12_ antagonist (TRACER [47]). One RCT in elective CABG patients was ongoing [48].

### Description of Included Studies and Quality Assessment

Table 1 provides details of the trials including baseline patient characteristics. Of the 9 included RCTs, five were single- [15, 16, 22, 23] or two-centre [21] RCTs that enrolled patients post elective CABG and compared clopidogrel plus ASA to ASA alone. One trial specified that ASA was not held pre-operatively [21]. ASA and clopidogrel were restarted post-operatively when chest tube bleeding was no longer significant, typically within 24–48 h. Patients were randomized either pre- [16] or post- [15, 21, 22] operatively. In one trial [23], patients were randomized on postoperative day 4 while on aspirin to clopidogrel add on but only if found to be aspirin resistant on aggregometry-based assessment.

The other four RCTs were all large multicentre trials enrolling patients with ACS that reported data separately for the subgroup of patients who underwent CABG [11, 17–19]. Of the ACS trials, 2 compared clopidogrel plus ASA to ASA alone [3, 20], and 2 compared higher intensity dual anti-platelet therapy with either ticagrelor [5] or prasugrel [4] plus ASA vs clopidogrel plus ASA. CREDO [20] enrolled patients who were referred for coronary angiography with symptomatic coronary artery disease (angina pectoris, positive stress test, or dynamic electrographic changes). CURE [3] enrolled patients with acute ischemic symptoms and either electrographic changes and/or elevation of cardiac enzymes to indicate myocardial necrosis, but excluded patients with ST elevation. Both PLATO [5] and TRITON-TIMI 38 [4] had similar inclusion criteria and allowed patients with either ST depression or ST elevation ACS. In TRITON-TIMI 38 [4] the coronary anatomy had to be defined and suitable for percutaneous coronary intervention (PCI) before randomization, hence a smaller percentage of patients progressed to CABG in this trial. In three of the included ACS trials [3–5], ASA and blinded second anti-platelet therapies were started shortly after presentation, whereas for CREDO [20] both randomized groups received dual anti-platelet therapy with clopidogrel for the first 28 days and then the control group received placebo for the remainder of the 1 year treatment period. For patients subsequently requiring CABG, the second anti-platelet therapy or corresponding placebo was typically held between 1 and 7 days pre-operatively, and then re-started post-operatively in the majority (62–76 %) of patients (Table 1) [11, 17, 19]. For CURE [19] and CREDO [18] outcome data was provided for all patients who underwent CABG. For PLATO outcome data were provided only for the 1261 of 1899 patients that underwent CABG who received study drug within 7 days prior to the procedure [17]. For TRITON-TIMI 38 all outcome data were only provided for the 346 of 485 randomized patients that underwent CABG who received at least one dose of study drug prior to the procedure [11]; additional outcome data on all randomized patients that underwent CABG were provided in a FDA presentation [49].

**Table 1:** Trial and Baseline Patient Characteristics, and Interventions

	Patients Randomised Post Elective CABG					Patients Randomized with ACS or symptomatic CAD - CABG Subgroup			
	Clopidogrel 300 then 75 mg/d vs placebo	Clopidogrel 75 mg/d	Clopidogrel 75 mg/d vs placebo	Clopidogrel 75 mg/d	Clopidogrel 75 mg/d	Clopidogrel 300 then 75 mg/d vs placebo^a^	Clopidogrel 300 then 75 mg/d vs placebo	Ticagrelor 180 then 90 mg bid vs Clopidogrel 300 then 75 mg/d	Prasugrel 60 then 10 mg/d vs Clopidogrel 300 then 75 mg/d
ASA Dose (mg/d)	325 then 81	100	162	100	300	325	75-325	75-100	75-162
Trial	Sun et al. [16]	Gao et al. [15]	CASCADE [21]	CRYSSA [22]	Gasparovic [23]	CREDO [18]	CURE [19]	PLATO [17]	TRITON- TIMI 38 [11]
*n* = CABG/total patients	*n* = 100/100	*n* = 249/249	*n* = 113/113	*n* = 300/300	*n* = 224/224	*n* = 83/2116	*n* = 2072/12562	*n* = 1261 (1899)/18624	*n* = 346 (485)/13608
Trial Characteristics									
No. of Centres	1	1	2	1	1	99	428	862	707
Enrolment period	Nov 2006 – Feb 2008	Dec 2007 – Dec 2008	May 2006 – Jul 2008	Dec 2006 – Oct 2009	Jun 2010 – Feb 2013	Jun 1999 – Apr 2001	Dec 1998 – Sep 2000	Oct 2006 – Jul 2008	Nov 2004 – Jan 2007
Treatment/Follow Up Post Randomization	30 (all)/49 (median) d	3 months (all)	12 months (all)	12 months (all)	6 months (all)	12 months (all)	9 months (mean)	224 days [7.5 months] (median)	14.5 months (median)
Median Time to CABG Post Randomization	(Randomized At Time of CABG)					n/r (index CABG instead of PCI)	25.5 days (IQR 12–70.5)	~20 days	100 days [50]
Treatment/Follow Up Post CABG	30 (all)/49 (median) d	3 months (all)	12 months (all)	12 months (all)	12 months (all)	~12 months (all)	~8 months (mean)	~200 days [6.7 months] (median)	~11.2 months (median)
Funding	Public/ Industry	Public	Public/Industry	n/r	n/r	Industry	Industry	Industry	Industry
Patients	*n* = 99	*n* = 224	*n* = 113	*n* = 300	*n* = 219	*n* = 83	*n* = 2072	*n* = 1261	*n* = 346
Mean Age (years)	65	59	67	59	65	61	64	64	61
% Male	90	83	89	74	75	74	70	79	77
BMI	31	26	28	26	29	29		27	n/r (28^b^)
Diabetes	35 %	40 %	29 %	0 % (excl)	38 %	22 %	27 %	32 %	28 %
Hypertension	70 %	59 %	50 %	46 %	96 %	75 %	61 %	68 %	64 %
Smoker	59.6 % (current/ former)	55.8 % (history of)	13.2 %	n/r	36 %	33 % (within 1 year)	64.5 % (current /former)	31.2 %	n/r (38 %^b^)
COPD				n/r		n/r			8.1 %
Dyslipidemia	76 %	38 %	88 %	56 %	96 %	71 %	n/r	54 %	n/r (56 %^b^)
Prev MI	39 %	47 %	n/r	36 %	n/r	37 %	36 %	20 %	n/r (18 %^b^)
Prev stroke/TIA	5.1 %	4.9 %	n/r	n/r	n/r	n/r	3.9 %	3.9 %/3.1 %	7.8 %
Periph Vasc Dz	5.1 %	n/r	n/r	n/r	n/r	6.2 %	9.7 %	7.6 %	7.0 %
Chronic Renal Disease	excl Cr >130 μM	n/r	n/r	excl Cr >177 μM	n/r	n/r	n/r	4.8 %	n/r (11 % CrCl < 60 mL/min^b^)
Mean LVEF	n/r	60 %		54 %	54 %	53 %			
CHF		0 % (excl)	20.3 %	n/r	n/r	7.3 %		4.1 %	10.7 %
Severe LV dysfunction	38 %(Gr 3 LV)	0 % (excl)	n/r	n/r	n/r	n/r	Excluded		3.9 % (EF ≤ 30 %)
	1 % (Gr 4 LV)								
Previous PCI	n/r	13 %		16 %	n/r	17 %	7.4 %	10 %	n/r
Previous CABG	n/r	0 % (excl)	0 % (excl)	n/r	n/r	8.5 %	4.6 %	1.5 %	2.9 %
3VD	54 %			n/r	77 %	n/r			
LM	24.2 %			n/r	48 %	n/r			
ST depression							54.2 %		
ST elevation							2.7 %	33.0 % (persistent)	
Abnormal ECG							95.2 %		
Diagnosis: UA							73.9 %		64.1 % (UA/NSTEMI)
Diagnosis: MI							26.1 % (all NSTEMI)		35.9 % (STEMI)
Abnormal ECG/enzymes							97.7 %		
CABG									
% Off-pump CABG	0 %	58.0 %	3.6 %	100 %	0 %				
% arterial conduits	100 %	100 %	100 %	100 %	93 %				
Grafts or ^a^diseased vessels	4.0	3.1	3.5 ± 0.7	3.2 ± 0.6	n/r			1–2 (31 %)	^a^1 (14.4 %)
								3–4 (60 %)	^a^2 (61.5 %)
								≥5 (8 %)	^a^3 (20.5 %)
Median (IQR) days to CABG (all)							25.5 (12–70.5)	~20	100(36)
CABG pre-d/c							49 %	57 %	4.3 %
Median (IQR) days to CABG pre-d/c							13 (8–21) vs 12 (8–19)		
CABG post-d/c							51 %	43 %	95.7 %
Median (IQR) days to CABG post d/c							67.5 (38–141) vs 73 (36–129)		
Days off study drug before CABG							17 (9–33)	≤2d (30.1 %) 3-5d (43.8 %) >5 (26.1 %)	≤2d (25.1 %) 3-5d (29.0 %) >5 (45.4 %)
Restarted treatment post CABG							76.1 % (66 never discontinued + 1451 resumed/1928; 78 data unavailable)	66.4 %	61.8 % (214/346); additional 21/173 prasugrel and 16/173 clopidogrel patients resumed open label clopidogrel
Days off study drug after CABG							10 (6–25)	<7d (35.9 %)	n/r
								7–14(16.8 %)	
								>14d (10.0 %)	

^a^In CREDO, both groups receive clopidogrel 75 mg/day for 28 days before control group changed to placebo from day 29 through 12 months

^b^Proportion of all 13,608 randomized patients when data not provided for CABG subgroup in TRITON-TIMI 38

*Abbreviations*: *ACS* acute coronary syndrome, *ASA* acetylsalicylic acid (aspirin), *BMI* body mass index, *CABG* coronary artery bypass grafting, *CAD* coronary artery disease, *CHF* congestive heart failure, *COPD* chronic obstructive pulmonary disease, *d/c* (hospital) discharge, *DM* diabetes mellitus, *Dx* diagnosis, *Dz* disease, *ECG* electrocardiogram, *EF* ejection fraction, *excl* excluded, *incl* included, *IQR* inter-quartile range, *LM* left main artery disease, *LV* left ventricle, *mg* milligram, *MI* myocardial infarction, *mM* millimolar, *n* number of patients, *no*. number, *n/r* not reported, *PCI* percutaneous coronary intervention, *periph* peripheral, *prev* previous, *pts* patients, *Rx* treatment, *sd* standard deviation, *TIA* transient ischemic attack, *vasc* vascular, *3VD* triple vessel disease, *UA* unstable angina, *vasc* vascular

For all trials, enrolled patients had a mean age around 60 years old, were predominantly male, and had the expected prevalence of various coronary risk factors (Table 1). 18–47 % of patients had previous myocardial infarction, and a small percentage (3–8 %) had previous stroke. A small percentage also had previously been treated with PCI or CABG. Patients with heart failure or severe left ventricular dysfunction were generally excluded. Some trials used off-pump surgery, and all patients received multiple grafts with at least one arterial conduit.

As shown in Table 1, in the 5 RCTs enrolling elective CABG patients, all patients were enrolled at the time of CABG and followed up for 12 months in two trials [21, 22], 6 months in one trial [23], 3 months in one trial [15], and a minimum of 30 days (median 49 days) in the fifth trial [16]. In 3 of the 4 RCTs enrolling ACS patients, CABG occurred at a median time of 20–100 days post-randomization [17, 19, 50] and patients were followed for a median of 6.7 to 11.2 months post CABG. Time to CABG and follow up duration was similar between groups in each trial. The fourth ACS trial enrolling patients expected to require PCI did not specifically report time to CABG but included only patients who were treated with CABG instead of PCI at study enrolment implying treatment shortly after randomization, and followed all patients for 12 months post randomization [18]. For all trials only clinical outcomes that occurred post CABG were included.

Study quality was relatively high (Table 2). Allocation was concealed in all trials except for one single-centre trial where it was unclear [15], and all studies except for three single-centre trials [15, 22, 23] blinded participants using placebos. All trials used intention-to-treat analysis, were not stopped early for benefit, and had <5 % (and for the large multi-centre trials ≤0.1 %) of randomized patients with missing outcome data.

**Table 2:** Quality assessment of included randomized controlled trials

Trial	Blinded	Concealed allocation	Intention to treat analysis	Not stopped early for benefit	< 5 % Randomized Patients with Missing Outcome Data
CURE [3, 19]	Yes	Yes (central randomization)	Yes	Yes	Yes (0.1 %: 13/12,562 overall; 0 % of CABG)
PLATO [5, 17]	Yes	Yes (central randomization)	Yes	Yes	Yes (0.03 %: 5/18,624 overall)
TRITON-TIMI 38 [4, 11]	Yes	Yes (central randomization)	Yes	Yes	Yes (0.1 %: 14/13,608 overall)
CREDO [18, 20]	Yes	Yes	Yes	Yes	Yes (0 %)
CRYSSA [22]	No	Yes	Yes	Yes	Yes (0.3 %: 1/300)
CASCADE [21]	Yes	Yes	Yes	Yes	Yes (0 %)
Gao 2010 [15]	No	Unclear	Yes	Yes	Yes (3.7 %: 9/249)
Sun 2010 [16]	Yes	Yes	Yes	Yes	Yes (1.0 %: 1/100)
Gasparovic 2014 [23]	No	Yes	Yes	Yes	Yes (2.2 %: 5/224)

### Quantitative Data Synthesis

**Fig. 2:**
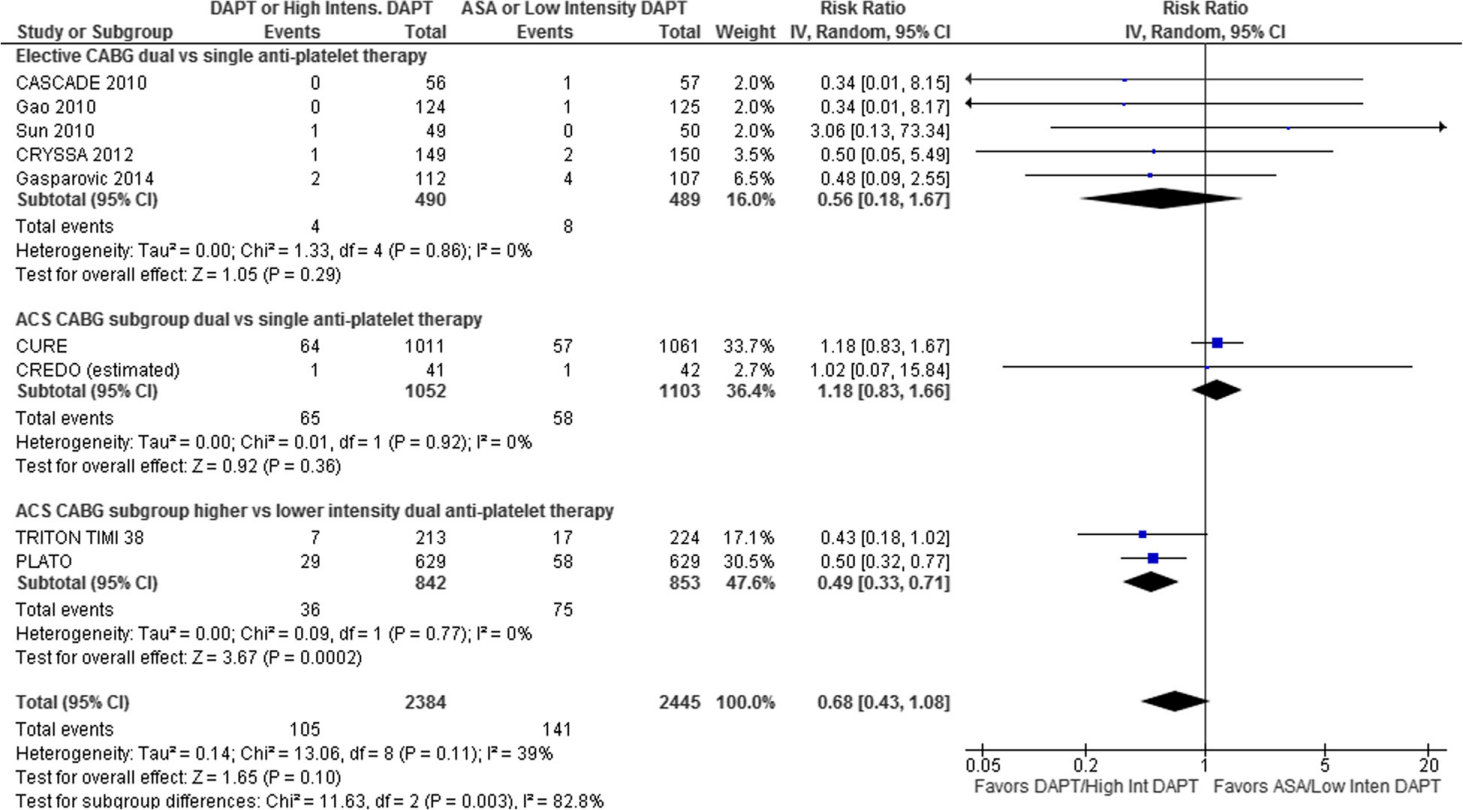
Forest plot for all-cause mortality. Individual and pooled risk ratios (RR) with 95 % confidence intervals (CI) for randomized controlled trials (RCTs) comparing dual anti-platelet therapy with clopidogrel and ASA to single anti-platelet therapy with ASA alone either after elective coronary artery bypass graft surgery (CABG) [15, 16, 21–23] or in patients with acute coronary syndrome (ACS) who subsequently underwent CABG [18, 19], and RCTs comparing higher to lower intensity dual anti-platelet therapy with either ticagrelor [17] or prasugrel [11] and ASA vs clopidogrel and ASA in patients with ACS who subsequently underwent CABG. The pooled RRs with 95 % CI were calculated using random-effects models both overall and for each subgroup. Weight refers to the contribution of each study to the overall pooled estimate of treatment effect. Each square and horizontal line denotes the point estimate and 95 % CI for each trial’s RR. The diamonds signify the pooled RR for all trials and each subgroup; the diamond’s centre denotes the point estimate and width denotes the 95 % CI. For CREDO, the number of all-cause deaths was estimated using the ratio of this outcome to the composite outcome for each randomized group reported in the main trial publication [20] because the ACS CABG publication [18] for this trial only provided composite outcomes

All-Cause Mortality (Fig. 2): Pooling data from all RCTs, there was no difference in all-cause mortality (RR 0.68, 95 % CI 0.43–1.08, *p* = 0.10) with some heterogeneity (*I*^*2*^ = 39 %). The clopidogrel plus ASA vs ASA RCTs showed no difference in all-cause mortality for either the elective CABG (RR 0.56, 95 % CI 0.18–1.67, *p* = 0.29) or the ACS CABG subgroups (RR 1.18, 95 % CI 0.83–1.66, *p* = 0.36). However, the ticagrelor or prasugrel vs clopidogrel RCTs showed significantly lower risk for all-cause mortality (RR 0.49, 95 % CI 0.33–0.71, *p* = 0.0002). There was no heterogeneity within any of the subgroups (*I*^2^ = 0 %). The differences between the clopidogrel plus ASA vs ASA elective CABG and ACS subgroups (either individually or combined), and the ticagrelor or prasugrel vs clopidogrel subgroup results were statistically significant (interaction p-value 0.002–0.007). Visual inspection of the funnel plot showed no evidence of asymmetry (data not shown).

**Fig. 3:**
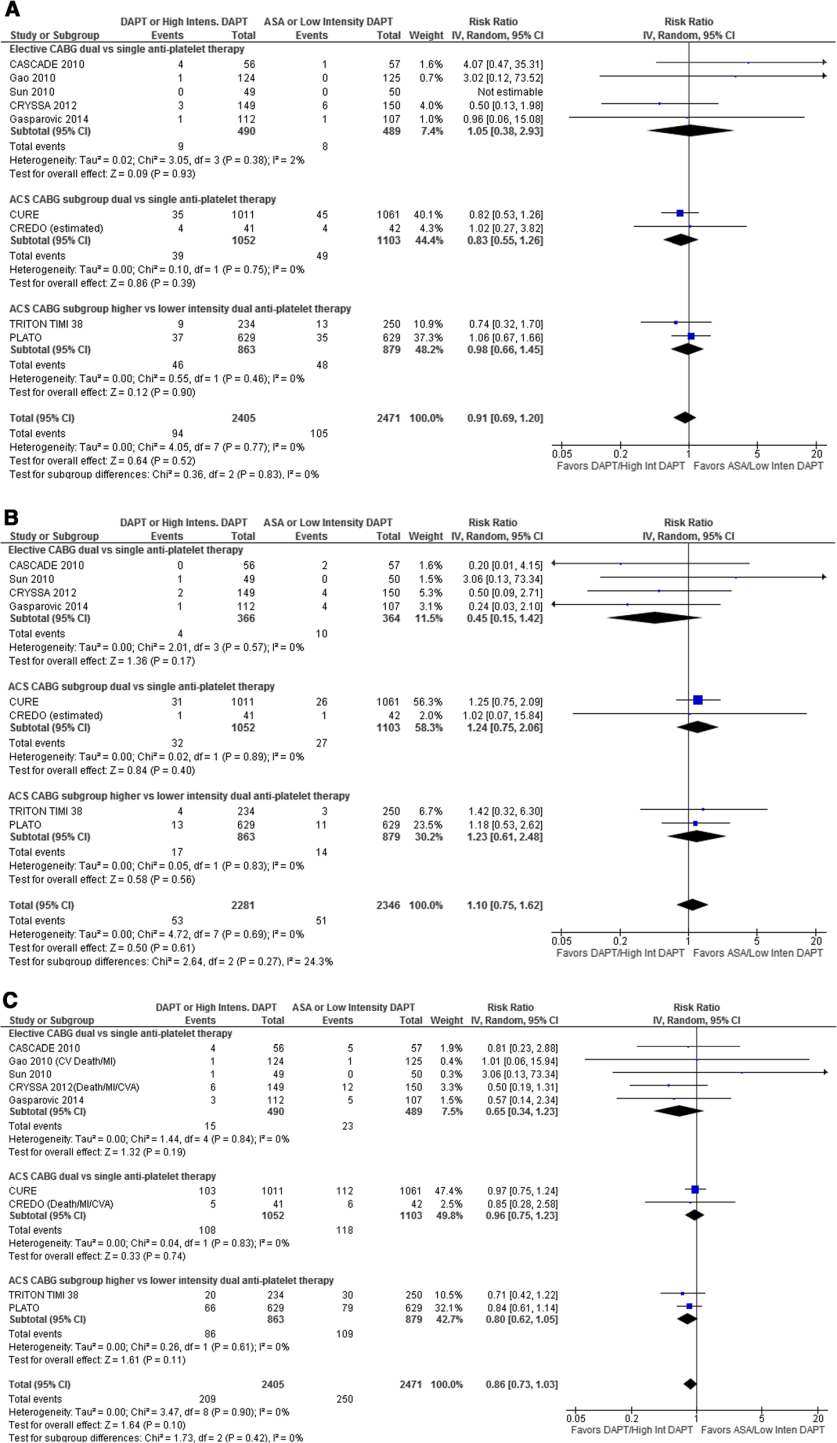
Forest plot for (**a**) myocardial infarction, (**b**) stroke, (**c**) composite outcome including cardiovascular mortality, myocardial infarction, and stroke. Individual and pooled risk ratios (RR) with 95 % confidence intervals (CI) for randomized controlled trials (RCTs) comparing dual anti-platelet therapy with clopidogrel and ASA to single anti-platelet therapy with ASA alone either after elective coronary artery bypass graft surgery (CABG) [15, 16, 21–23] or in patients with acute coronary syndrome (ACS) who subsequently underwent CABG [18, 19], and RCTs comparing higher to lower intensity dual anti-platelet therapy with either ticagrelor [17] or prasugrel [11] and ASA vs clopidogrel and ASA in patients with ACS who subsequently underwent CABG. The pooled RRs with 95 % CI were calculated using random-effects models both overall and for each subgroup. Weight refers to the contribution of each study to the overall pooled estimate of treatment effect. Each square and horizontal line denotes the point estimate and 95 % CI for each trial’s RR. The diamonds signify the pooled RR for all trials and each subgroup; the diamond’s centre denotes the point estimate and width denotes the 95 % CI. For CREDO, the number of myocardial infarctions and strokes was estimated using the ratio of this outcome to the composite outcome for each randomized group reported in the main trial publication [20] because the ACS CABG publication [18] for this trial only provided composite outcomes. For the composite outcome reported in Panel C, only all-cause mortality was available for CRYSSA 2012 [22] and CREDO [18], and only cardiovascular death and myocardial infarction for Gao 2010 [15]

Other Clinical Outcomes: Neither dual vs single nor higher-intensity vs lower-intensity dual anti-platelet therapy resulted in decreased myocardial infarction (RR 0.91, 95 % CI 0.69–1.20, *p* = 0.52; Fig. 3a) or stroke (RR 1.10, 95 % CI 0.75–1.62, *p* = 0.61; Fig. 3b) with similar non-significant treatment effects in the dual vs single anti-platelet and higher-intensity vs lower-intensity DAPT subgroups (*I*^2^ = 0 %). Likewise the composite outcome of death from cardiovascular causes (all-cause mortality for CRYSSA [22] and CREDO [18]), myocardial infarction, and stroke (only cardiovascular death and myocardial infarction for Gao 2010 [15]) remained non-significant (Fig. 3c): RR 0.86, 95 % CI 0.73–1.03, *p* = 0.10, with no differences in results between subgroups (*I*^2^ = 0 %).

**Fig. 4:**
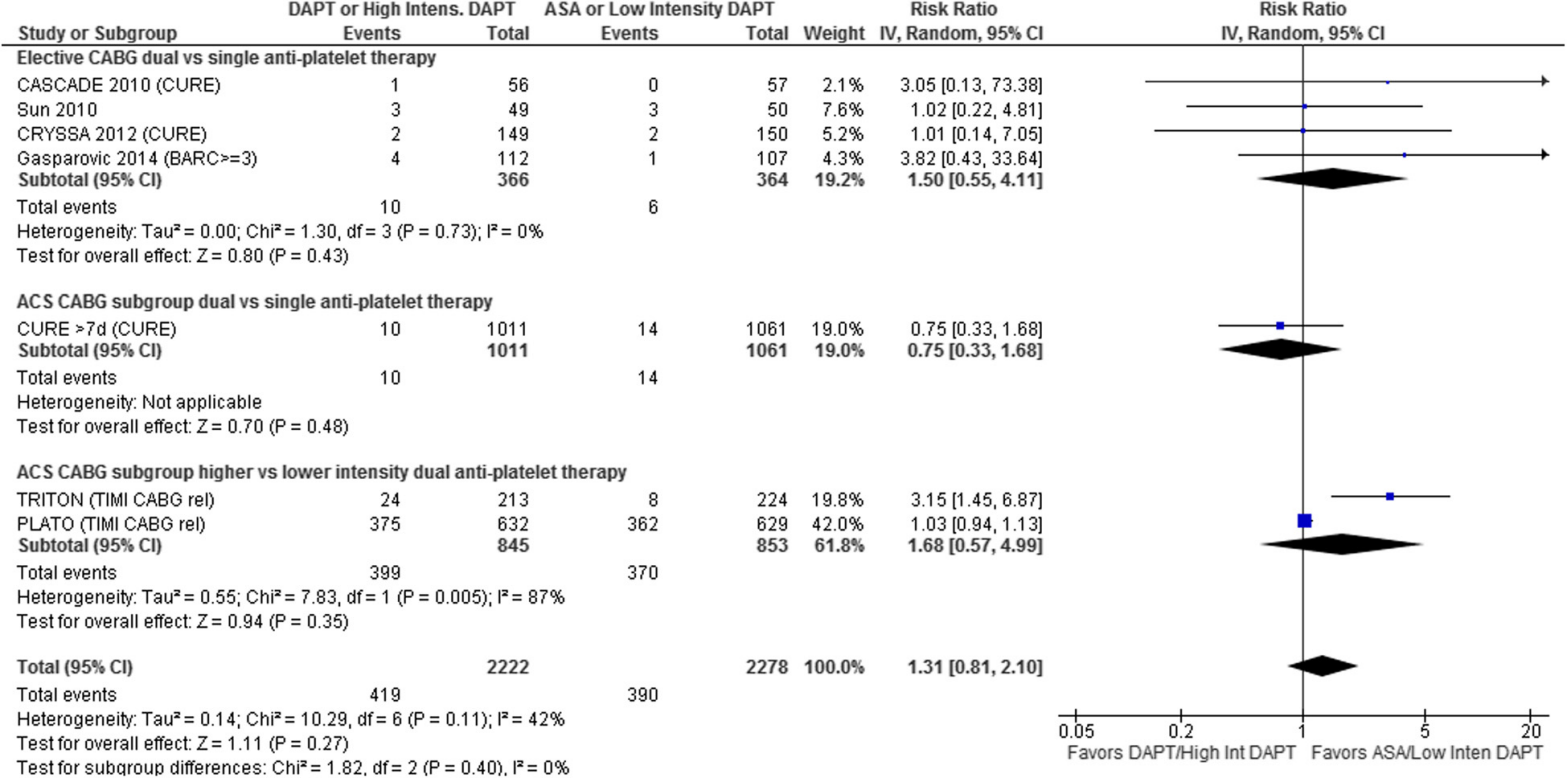
Forest plot for major bleeding. Individual and pooled risk ratios (RR) with 95 % confidence intervals (CI) for randomized controlled trials (RCTs) comparing dual anti-platelet therapy with clopidogrel and ASA to single anti-platelet therapy with ASA alone either after elective coronary artery bypass graft surgery (CABG) [16, 21–23] or in patients with acute coronary syndrome (ACS) who subsequently underwent CABG [19], and RCTs comparing higher to lower intensity dual anti-platelet therapy with either ticagrelor [17] or prasugrel [11] and ASA vs clopidogrel and ASA in patients with ACS who subsequently underwent CABG. The pooled RRs with 95 % CI were calculated using random-effects models both overall and for each subgroup. Weight refers to the contribution of each study to the overall pooled estimate of treatment effect. Each square and horizontal line denotes the point estimate and 95 % CI for each trial’s RR. The diamonds signify the pooled RR for all trials and each subgroup; the diamond’s centre denotes the point estimate and width denotes the 95 % CI. The clopidogrel plus ASA vs ASA trials used either CURE trial [19, 21, 22], Bleeding Academic Research Consortium (BARC) Type 3–5 [23] (all BARC Type 3 for this trial [i.e., no CABG-related {Type 4} or fatal {Type 5} bleeding]), or similar [16] criteria for major bleeding and reported events that were either non-CABG related [16, 21–23] or more than 7 days post CABG [19]. The higher-intensity vs lower-intensity dual anti-platelet PLATO [17] and TRITON-TIMI 38 trials reported bleeding using TIMI criteria and included CABG related bleeding which made up the vast majority of the bleeding events (non-CABG related bleeding data was not available for the ACS CABG patients in these RCTs). If TIMI criteria are used for the CURE trial [19], the pooled results are essentially unchanged (RR 1.25, 95 % CI 0.71–2.19, *p* = 0.43; *I* ^*2*^ = 48 %). Excluding TRITON TIMI 38 [11] from the pooled results eliminates the heterogeneity (*I* ^*2*^ = 0 %)

Bleeding: The clopidogrel plus ASA vs ASA trials that reported bleeding used CURE trial [19, 21, 22], Bleeding Academic Research Consortium (BARC) Bleeding Definitions [51] Type 3–5 [23] or similar [16] criteria for major bleeding and reported events that were either non-CABG related [16, 21–23] or more than 7 days post CABG [19]. The higher-intensity vs lower-intensity DAPT trials reported bleeding using TIMI criteria and included both CABG-related and non-CABG-related bleeding. One of these trials specifically stated that “nearly all bleeding events occurred within 24 h post-CABG” [17]. Overall, major bleeding was not significantly increased (RR 1.31, 95 % CI 0.81–2.10, *p* = 0.27; Fig. 4) with some heterogeneity (*I*^2^ = 42 %) but no differences between subgroup results. The heterogeneity was due entirely to the significantly higher bleeding rate reported for TRITON-TIMI 38 (*I*^2^ = 0 % when the results of this trial are excluded).

## Discussion

The results from this analysis suggest that DAPT resumption with higher intensity P2Y_12_ antagonists (prasugrel or ticagrelor), but not clopidogrel reduces all-cause mortality in ACS patients who have undergone CABG. However, these results are based on post-randomization subsets from single RCTs where DAPT was initiated prior to CABG, with the intention of continuing therapy for one year post revascularization. Although it would have been invaluable to compare the outcomes in patients who continued DAPT vs. those in whom therapy was not resumed post-CABG, these data were not available. Likewise, there are no RCTs wherein ACS patients were randomized to receive DAPT vs. ASA following CABG. Finally, the number of patients enrolled in elective CABG RCTs was considerably smaller, and the pooled results of these RCTs comparing exclusively clopidogrel plus ASA to ASA alone post operatively demonstrated no differences in outcomes.

Among the higher-intensity P2Y_12_ antagonist RCTs, it is important to highlight that in TRITON-TIMI 38 prasugrel was only initiated after the coronary anatomy was defined and PCI was planned [11]. The trial was not intended to recruit patients destined for CABG, although in the minority of patients who did undergo CABG, the prasugrel group yielded a significant reduction in mortality. Prasugrel was associated with an increased risk of major bleeds which was primarily CABG related. A subsequent analysis by the trial authors indicated that prasugrel-treated patients received higher platelet but not red blood cell transfusions [50].

Ticagrelor is a novel P2Y_12_ antagonist, with a reversible mechanism of action. It provides more effective and reliable inhibition of platelet activity compared to clopidogrel [52], and recent data suggest that it may also have a unique pleiotropic effect to augment adenosine bioavailability [53]. In the PLATO trial, randomization for 1-year to DAPT with ticagrelor vs. clopidogrel reduced all-cause mortality in the entire cohort by 20 %; the magnitude of benefit was particularly striking in those who underwent CABG (of whom approximately 66 % were restarted on study drug), where an approximate 50 % reduction in mortality was observed [17]. Interestingly, CABG related major bleeding, defined by various criteria was not different between ticagrelor vs. clopidogrel arms, and neither were the rate of transfusion or mean chest tube drainage. However, non-CABG related major bleeds were slightly higher in the ticagrelor vs. clopidogrel treated patients in all patients [5]. (Non-CABG related major bleed data was unfortunately unavailable for the CABG subset [17]). Since non-CABG related bleeding is likely the safety outcome of interest outside of the immediate perioperative period, surgeons must weigh the bleeding risk relative to the major reduction in all-cause mortality in choosing whether or not to resume DAPT with ticagrelor [54]. Recent data suggest that the adjusted hazard ratio for bleeding with ticagrelor does not increase with age [55].

The present analysis does not provide insight regarding what types of ACS patients are more likely to benefit from DAPT post CABG. It may be hypothesized that patients with diffuse disease (diabetes and chronic kidney disease for example), may be more likely to derive ongoing ischemic benefit by virtue of reduction in new atherothrombotic events. Likewise, analysis of benefit as a function of complexity of lesions (SYNTAX score for example) has not been conducted. Individualizing therapy based on residual platelet reactivity while on treatment which can vary widely between patients may help to better identify patients who would benefit from higher intensity anti-platelet therapy. For example, the most recently published trial included in this meta-analysis [23] attempted to do this by randomizing only patients to DAPT who still had high residual platelet reactivity while on ASA. Further studies aimed at better refining the subgroups that are more likely to derive the greatest ischemic benefit, and yield the lowest bleeding signal need to be performed.

Our systematic review and meta-analysis is the first to include both RCTs enrolling patients undergoing elective CABG and subgroups of patients presenting with ACS who subsequently require CABG. Furthermore, we also included RCTs that employed both higher- and lower-intensity dual antiplatelet therapies. Because results from these different types of RCTs may differ, we separated them into subgroups of RCTs for each analysis to highlight potential differences. Previous systematic reviews have been more restrictive either including only studies enrolling elective CABG patients [56–58], comparing lower intensity dual antiplatelet therapy with clopidogrel plus ASA to ASA [59], comparing dual or single antiplatelet therapy with clopidogrel to ASA [60, 61], or comparing higher- to lower-intensity dual anti-platelet therapy [62, 63]. These systematic reviews are in addition to those evaluating the use of dual anti-platelet therapy at the time of CABG [64–71]. We included all RCTs in any of these systematic reviews that met inclusion criteria for the current review.

### Study Limitations

Although we used rigorous systematic review and meta-analytic methods consistent with PRISMA guidelines including a reproducible and comprehensive literature search strategy, clearly defined inclusion criteria, duplicate citation review, data abstraction, and quality assessment of individual studies, and a pre-defined analysis plan, we pooled results from studies that employed different inclusion/exclusion criteria, interventions, and follow up periods. In particular, we pooled results from trials comparing dual vs single antiplatelet therapies (clopidogrel plus ASA vs ASA) with trials using different higher intensity dual platelet therapies (using ticagrelor or prasugrel) vs lower intensity dual platelet therapies (using clopidogel). We also pooled results from trials enrolling elective CABG patients with ACS patients who were randomized to dual anti-platelet therapy groups and then subsequently required CABG. Recognizing these differences, we presented pooled results separately for each of these subgroups within each analysis. The small size of elective CABG patient trials resulted in few events highlighting the paucity of currently available data (as well as upcoming data from ongoing registered trials listed in Additional file 1: Table S1) in this specific patient population, making it difficult to assess either the benefits, in terms of reducing adverse cardiac events, or the harms, in terms of increased bleeding. Follow up duration was variable ranging from 1.5 to 12 months for the elective CABG patients trials, and 6–12 months for the trials enrolling ACS patients who subsequently required CABG. The majority of the patient data was obtained from the large multicentre trials enrolling patients with ACS in which only a subgroup of patients underwent CABG [11, 17–19], at various time points, up to a median of 100 days post randomization in one trial [50]. Although separate subgroup data for the patients treated with CABG have been reported in the ACS RCTs, the decision to undergo CABG is a post randomization event occurring at variable times post randomization. This decision can thus be influenced by randomized group resulting in potential baseline imbalances between intervention and control groups within the subgroup, though the baseline characteristics reported for the subgroups undergoing CABG were balanced in these trials [11, 17–19]. Furthermore in some of these trials, complete outcome data are only reported for some of the patients who underwent CABG [11, 17] and these patients may be lower risk than CABG ACS patients treated outside of clinical trials. Except for bleeding events in CURE which were reported greater than 7 days post CABG, bleeding events in the other ACS trials though reported postoperatively also included events related to the operation. For example, bleeding events in TRITON-TIMI 38 and PLATO included all post-operative events and one of these reports stated that “nearly all bleeding events occurred within 24 h post-CABG” [17]. Finally, for the subgroups of patients with acute coronary syndrome who underwent CABG, we analyzed the patients on an intention to treat basis; only 62–76 % of the patients in the three largest trials [11, 17, 19] resumed dual antiplatelet therapy post operatively. Incomplete resumption of assigned treatment will reduce apparent treatment and adverse effects. Only TRITON-TIMI 38 provided on-treatment data and then only for mortality [11]. Conducting an on-treatment analysis, if these data were available for all trials, may provide better efficacy data; however, this would involve incorporating a further post-randomization event potentially leading to non-comparable groups with dissimilar baseline characteristics. For ACS patients the timing of DAPT treatment is likely more complex since when DAPT is discontinued preoperatively (to maximize the ACS benefit and to minimize bleeding) and when DAPT is resumed postoperatively (to maximize post-operative benefits) may both potentially affect outcomes.

## Conclusions

Based on the available but limited RCT data, resumption of anti-platelet therapy post operatively with higher intensity DAPT (prasugrel or ticagrelor with ASA) but not lower intensity DAPT (clopidogrel and ASA) appears to reduce all-cause mortality by about 50 % in patients with ACS who undergo CABG. With the caveat that the data are primarily based on retrospective subgroup analysis from single RCTs of ACS patients who progress to CABG, the net clinical benefit (efficacy vs. bleeding) appears to favour the use of ticagrelor in these patients. No significant benefits or harms were detected for DAPT after elective CABG, however, few such patients have been studied in randomized trials. The limited RCT data suggest that large prospective RCTs evaluating the use of DAPT post-CABG are urgently needed to provide more definitive guidance for clinicians.

## Additional file

Additional file 1:
**Description of Excluded RCTs.** (DOC 63 kb)

## Abbreviations

ACS: Acute coronary syndrome
ASA: Acetylsalicylic acid
CABG: Coronary artery bypass graft surgery
CI: Confidence interval
DAPT: Dual antiplatelet therapy
MI: Myocardial infarction
NSTEMI: Non-ST segment elevation myocardial infarction
PCI: Percutaneous coronary intervention
RCT: Randomized controlled trial
RR: Risk ratio
STEMI: ST-elevation myocardial infarction
