# Supplementary material for: Should dual antiplatelet therapy be used in patients following coronary artery bypass surgery? A meta-analysis of randomized controlled trials
Source: BMC Surg. 2015 Oct 14;15:112. doi: 10.1186/s12893-015-0096-z (PMC4605093; doi:10.1186/s12893-015-0096-z)
Supplement: Additional file 1: — Description of Excluded RCTs. (DOC 63 kb) [file 12893_2015_96_MOESM1_ESM.doc]

**Additional file 1: Description of Excluded RCTs**

| **Excluded Trial** | **Patient** **Inclusion** | **Number of Pts / Centres** | **Intervention** | **Control** | **Follow Up Duration** | **Outcomes** | **Reason for Exclusion** |
| --- | --- | --- | --- | --- | --- | --- | --- |
| **Post CABG** | | | | | | | |
| Saw 2015 [29] | Elective CABG | 70 / single | Ticagrelor 90 bid + ASA 81 mg/d | ASA 81 mg/d | 12 months | Graft patency; adverse cardiovascular events; bleeding | - Published only as conference abstract with no reported outcome events |
| Thopte 2014 [30] | Elective CABG | 74 / single | Clopidogrel 75 + ASA 150 mg/d | ASA 150 mg/d | 3 months | Graft patency; adverse cardiovascular events; bleeding | - Published only as conference abstract with no reported outcome events |
| Wang 2013 [28, 31] | Off Pump CABG | 60 / single | Clopidogrel 75 + ASA 100 mg/d | ASA 100 mg/d | Hospital discharge (mean 13d post op) | Platelet aggregation assays | -No reported events |
|  |  |  |  |  |  | “Cardiac events” (myocardial infarction, heart failure, death); bleeding | -Only short term intervention and follow up |
| Gao 2009 [24] | Elective CABG | 197 / single | Clopidogrel 75 mg/d + ASA 100 mg/d | Clopidogrel 75 mg/d | 12 months | Graft patency; mortality; bleeding | -No mortality, bleeding or other clinical events |
| Mujanovic 200 [26] | Elective Off Pump CABG | 20 / single | Clopidogrel 150 then 75 mg/d + ASA 100 mg/d | ASA 100 mg/d | 3 months | Graft patency; mortality; bleeding. | -No reported events |
| Kayacioglu 2008 [25] | Elective CABG with platelets >450 | 40 / single | Clopidogrel 75 mg/d + ASA 300 mg/d | ASA 300 mg/d | 6 months | Graft patency by cath in those with positive stress tests | -No reported clinical events (7/20 ASA only patients had positive stress test tests and 5/7 had new stenoses) vs 0/20 clopidogrel + ASA |
| Nielsen 2007 [27] | Elective Off Pump CABG | 29 / single | Clopidogrel 75 mg/d x 30d + ASA 150 mg/d | ASA 150 mg/d | 2 months | Thrombo-elasto-graphy and platelet inhibition studies; clinical outcomes | -No reported clinical events |
| Suwalski 2012 [34] | Elective Off Pump CABG | 50 / single | Clopidogrel 75 mg/d | ASA 150 mg/d | 6 months | Mortality, revasc, stroke, bleeding | -Single anti-platelet therapy |
|  |  |  |  |  |  |  | -Abstract only |
| Tetik 2010 [35] | Elective CABG | 50 / single | Clopidogrel 75 mg/d | ASA 300 mg/d | 6 months | Platelet aggregation assays, mortality, CV events, bleeding | -Single anti-platelet therapy |
| Lim 2004 [33, 36, 37] | Elective CABG | 54 / single | Clopidogrel 75 mg/d | ASA 100 or 325 mg/d (2 groups) | 5 days | Platelet aggregation | -Single anti-platelet therapy |
| David 1999 [32] | Elective CABG | 62 / single | Clopidogrel 50, 75, or 100 mg/d (3 groups) | Ticlopidine 250 mg bid | 28 days | Bleeding time and platelet aggregation | -Single anti-platelet therapy |
| Rafiq 2012 [48] | Hypercoag-ulable Elective CABG | (250) / single | Clopidogrel 75 ng/d + ASA 75 mg/d | ASA 75 mg/d | 3 months |  | Ongoing NCT 01046942 |
| **Acute Coronary Syndrome Requiring CABG** | | | | | | | |
| CURRENT-OASIS 7 2010 [44] | ACS w/ EKG or enzymes planned PCI | 25,086 / multiple (CABG n=~2000 [8%]) | Clopidogrel 600 then 150 mg/d x 6d then 75 mg/d (also 300-325 vs 75-100 mg/d ASA random-ization) | Clopidogrel 300 then 75 mg/d (also 300-325 vs 75-100 mg/d ASA random-ization) | 30 days | Mortality, MI, stroke, bleeding | -Only short term intervention and follow up |
|  |  |  |  |  |  |  | -no post CABG intervention |
|  |  |  |  |  |  |  | -no separate data for CABG subgroup |
| JUMBO-TIMI 26 2005; Phase 2 [42] | Elective or urgent PCI | 904 / 80 | Prasugrel 40-60 then 7.5-15 mg/d + ASA | Clopidogrel 300 then 75 mg/d + ASA | 30 days | Bleeding, mortality, MI, stroke | -Only short term intervention and follow up |
|  |  |  |  |  |  |  | -no separate data for CABG subgroup |
| DISPERSE-2 2007; Phase 2 [39] | NSTE-ACS | 990 / 152 (CABG n=84) | Ticagrelor 90-180 mg bid + ASA | Clopidogrel 300 then 75 mg/d + ASA | 12 weeks | Bleeding, mortality, MI, stroke | -no separate data for CABG subgroup |
| Prior CABG (median 9y) 2013, PLATO Substudy [46] | ACS | 1133 (of 18,613) / 862 | Ticagrelor 180 then 90 mg bid + ASA | Clopidogrel 300-600 then 75 mg/d + ASA | 360 days | Mortality, MI, stroke, bleeding | -no CABG during study period |
| Prior CABG 2001, CAPRIE Substudy [45] | Recent MI or CVA, or PVD | 1480 (of 19,185) / 384 | Clopidogrel 75 mg/d | ASA 325 mg/d | 1.6 years (mean) | Mortality, MI, stroke, bleeding | -Only single anti-platelet therapy |
|  |  |  |  |  |  |  | -no CABG during study period |
| TRILOGY ACS 2012 [41] | ACS without planned revasc. | 9326 / multiple | Prasugrel 30 then 5-10 mg/d + ASA | Clopidogrel 300 then 75 mg/d + ASA | 14.8 months (median) | Mortality, MI, stroke, bleeding | -no separate data for CABG subgroup |
| CHARISMA 2006 [38] | CV disease or multiple risk factors | 15,603 / 768 | Clopidogrel 75 mg/d + ASA 75-162 mg/d | ASA 75-162 mg/d | 28 months (median) | Mortality, MI, stroke, bleeding | -no separate data for CABG subgroup |
| CABG Subgroup | STEMI treated with fibrinolysis | 136 (3491) / 319 | Clopidogrel 300 then 75 mg/d + ASA 150-325 then 75-162 mg/d | ASA 150-325 then 75-162 mg/d | 30 days | Mortality, MI, stroke, bleeding | -median clopidogrel treatment 4d (prior to CABG); only 8/136 patients resumed post CABG |
| CLARITY-TIMI 28 2007 [43] |  |  |  |  |  |  |  |
| COMMIT 2005 [40] | AMI (93% STEMI) without planned PCI | 45,852 / 1250 | Clopidogrel 75 mg/d + ASA 162 mg/d | ASA 162 mg/d | 28 days | Mortality, MI, stroke, bleeding | -no separate data for CABG subgroup |
| TRACER 2012 [47] | NSTE-ACS | 1312 (12,944) / 818 | Voraxapar (PAR-1 antagonist) + clopidogrel + ASA | Clopidogrel + ASA | 1 year | Mortality, MI, stroke, bleeding | -triple anti-platelet therapy with non-P2Y_12_ antagonist |
